# Latissimus dorsi flap for breast reconstruction: a large single-institution evaluation of surgical outcome and complications

**DOI:** 10.1007/s00404-023-07186-3

**Published:** 2023-08-16

**Authors:** Maggie Banys-Paluchowski, Laura Brus, Natalia Krawczyk, Sophie Valeria Kopperschmidt, Maria Luisa Gasparri, Nana Bündgen, Achim Rody, Lars Hanker, Franziska Hemptenmacher, Peter Paluchowski

**Affiliations:** 1 Department of Obstetrics and Gynecology, University Hospital of Schleswig Holstein, Campus Lübeck, Lübeck, Germany; 2 Department of Gynecology and Obstetrics and Breast Cancer Center, Regio Klinikum Pinneberg, Pinneberg, Germany; 3 Department of Gynecology and Obstetrics, University of Düsseldorf, Düsseldorf, Germany; 4 Practice for Obstetrics and Gynaecology, Dr. Ganders and Dr. Hemminger, Hamburg, Germany; 5 Department of Gynecology and Obstetrics, Ospedale Regionale di Lugano EOC, Lugano, Switzerland; 6 Faculty of Biomedical Sciences, Università Della Svizzera Italiana, Lugano, Switzerland

**Keywords:** Breast cancer, Breast reconstruction, Thoracic reconstruction, Latissimus dorsi flap, Autologous reconstruction

## Abstract

**Purpose:**

The use of autologous tissues is considered gold standard for patients undergoing breast reconstruction and is the preferred method in the post-radiation setting. Although the latissimus dorsi flap (LDF) has been replaced by abdominal flaps as technique of choice, it remains a valuable option in several specific clinical situations and its use has been regaining popularity in recent years. In this work, we present an 18-year retrospective analysis of a single-institution single-surgeon experience with LDF-based reconstruction with focus on early complications and reconstructive failures.

**Methods:**

Hospital records of all patients undergoing breast surgery for any reason in the Certified Breast Cancer Center, Regio Klinikum Pinneberg, Germany between April, 1st 2005 and October, 31st 2022 were reviewed. 142 consecutive LDF-based reconstructive procedures were identified. Detailed information was gathered on patient characteristics, treatment-related factors, and complications.

**Results:**

One hundred forty patients (139 female, 1 male) received 142 LDF-based surgeries. The flap was used mainly for immediate breast reconstruction with or without implant (83% of patients), followed by defect coverage after removal of a large tumor (7%), implant-to-flap conversion with or without placement of a new implant (6%), and delayed post-mastectomy reconstruction (4%). The use of LDF decreased between 2005 and 2020 (2005: 17, 2006: 13, 2007: 14, 2008: 16, 2009: 5, 2010: 9, 2011: 8, 2012: 3, 2013: 10, 2014: 8, 2015: 8, 2016: 7, 2017: 7, 2018: 4, 2019: 4, 2020: 2, 2021: 6, 2022: 4). Surgery was performed for invasive breast cancer in 78%, ductal carcinoma in situ in 20% and other reasons such as genetic mutation in 1% of patients. Ipsilateral radiation therapy was received by 12% of patients prior to LDF surgery and by 37% after the surgery. 25% of patients were smokers. The median duration of surgery, including all procedures conducted simultaneously such as e.g., mastectomy, axillary surgery, or implant placement, was 117 min (range 56–205). Patients stayed in the hospital for a median of 7 days (range 2–23 days). The most common complication was seroma (26%), followed by wound dehiscence (8%), surgical site infection (7%), partial skin and/or nipple necrosis of any size (7%) and hematoma requiring surgical evacuation (2%). 19% of all patients required seroma aspiration or drainage, mostly at the donor site and performed under ultrasound guidance in the ambulatory setting. Flap loss due to necrosis occurred in 2% of patients.

**Conclusions:**

Latissimus dorsi flap is a well-established surgical technique commonly used for immediate breast reconstruction as well as defect coverage in locally advanced breast cancer. To the best of our knowledge, this is one of the largest single-surgeon analyses of early complications in patients receiving LDF. As expected, seroma was the most common complication observed in nearly one third of patients and requiring a therapeutic intervention in every fifth patient. Serious adverse events occurred rarely, and flap loss rate was very low.

**Supplementary Information:**

The online version contains supplementary material available at 10.1007/s00404-023-07186-3.

## What does this study add to the clinical work

**Table:** 

This analysis confirms latissimus dorsi flap-based breast surgery as a robust technique with a low complication rate. The most common complication is the development of a seroma and flap loss is a rare occurrence.	

## Introduction

The use of autologous tissues is considered gold standard for patients undergoing breast reconstruction, and is the preferred method in the post-radiation setting [1, 2]. Several procedures are available including pedicled flaps such as latissimus dorsi flap (LDF) and transverse rectus abdominis myocutaneous flap (TRAM), as well as free flap requiring microvascular anastomoses like the deep inferior perforator flap (DIEP). Some of these, like LDF, may be combined with implants to provide adequate volume and symmetry. In patients with limited soft tissue following mastectomy, breast reconstruction may involve several steps, e.g., tissue expander placement before positioning a permanent implant, surgery of the contralateral breast, or nipple and areola reconstruction.

While LDF is one of the oldest described muscle flaps, it did not gain wide recognition until the 1970s [3]. In the following years, numerous variations were introduced, such as de-epithelialized flap for volume replacement and extended flap including lumbar fat to maximize tissue amount [4, 5]. Although LDF has been replaced by abdominal flaps as autologous tissue of choice for breast reconstruction in most countries, it remains a reliable alternative and a valuable option in several specific clinical situations [6]. Its advantages are the robust vascular supply making flap necrosis a rare event, and, in case of combination approach, lower rates of capsular contracture compared to implant-only based reconstruction [7–10].

Although the use of LDF is regaining popularity in recent years [7, 8], evidence on complication rates and outcomes is limited. The 2015 German S3 guideline on breast reconstruction with autologous tissue identified only 22 articles on LDF-based reconstruction that reported on complication rates, the largest including 78 flaps [11]. In this work, we present an 18-year retrospective analysis of a single-institution single-surgeon experience with LDF-based reconstruction with a focus on early complications and reconstructive failures.

## Material and methods

In this retrospective study, clinical records of all patients undergoing breast surgery for any reason in the Certified Breast Cancer Center, Regio Klinikum Pinneberg, Germany between 01.04.2005 and 30.10.2022 were reviewed. 142 latissimus dorsi flap (LDF) reconstructive procedures were identified. The hospital electronic record was then reviewed for patient-, tumor- and treatment-related information. The following information were included in the database: age, body mass index (BMI), smoking habits, concomitant diseases and medication, timing of reconstruction, previous breast surgery, type of breast and axillary surgery, surgery duration, simultaneous use of an implant, complications, and secondary surgeries were included in the database. The protocol version 1.1 of this retrospective analysis has been reviewed by the Ethical Committee of the Medical Board Schleswig–Holstein (27/02/19).

### Statistical analysis

Chi-squared test and Fisher’s exact test were used to evaluate the relationship between complication rates and surgical parameters. All reported p-values are two-sided. Statistical analysis was performed by SPSS, version 18 (SPSS Inc., Chicago, IL, USA).

## Results

**Fig. 1:**
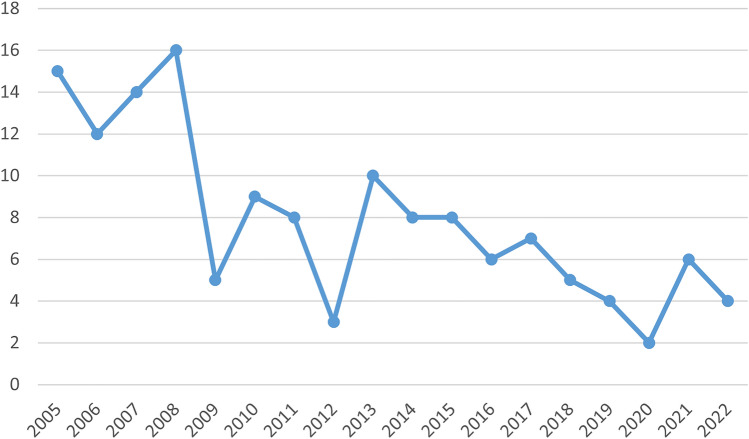
Changes in the number of LDF-based surgeries performed per year in the period analyzed (2005–2022)

Patient characteristics, diagnosis, surgical setting, and previous and subsequent oncological treatments for the 142 LDF procedures performed in 140 patients are presented in Table 1. All procedures were performed by the same surgeon (P.P.). All procedures were performed as open surgery and neither endoscopic nor robotic approach was used. The humeral insertion of the latissimus dorsi muscle was transected in all cases [12]. All patients received single-shot antibiotic prophylaxis. Extended antibiotic prophylaxis was not used. Mean age was 61 and the median follow-up was 38.4 months. 25% of patients were smokers at time of surgery. Invasive breast cancer was the main leading diagnosis accounting for 78% of the cases (Table 1). 139 patients were female and only 1 patient was male. 138 patients received a unilateral LDF surgery and in two cases, LDF was used for both sides, albeit not simultaneously: (1) 38-year-old patient undergoing a mastectomy with immediate LDF- and implant reconstruction for locally advanced breast cancer was diagnosed with a contralateral multicentric breast cancer 8 years later and received skin-sparing mastectomy with LDF and implant; (2) 83-year-old patient undergoing a radical mastectomy for an ulcerated 19 cm large tumor with LDF coverage was diagnosed with extended contralateral cutaneous metastases 4 months later and underwent a mastectomy with wide-excision of all skin manifestations combined with LDF defect coverage. The number of procedures per year decreased during the period analyzed from 41 surgeries performed between 2005 and 2007 to 12 between 2020 and 2022 (Fig. 1, Supp. Table 1).

**Table 1:** Clinical characteristics of all LDF procedures included in the analysis

	All procedures	Immediate reconstruction^1^	Delayed reconstruction^2^	Defect coverage after removal of a large tumor	Revision or implant-to-flap conversion^3^	p-value
Total	142	118	5	10	9	
Age (median [range])	61 (23–84)	50 (23–73)	53 (50–62)	74 (39–83)	49 (45–84)	< 0.001
BMI (median [range])	24 (17–42)	23 (17–42)	27 (25–27)	21 (19–38)	25 (22–38)	0.509
Diagnosis^4^						
Invasive cancer	111 (78%)	93 (79%)	4 (80%)	10 (100%)	5 (56%)	0.005
Ductal carcinoma in situ	29 (20%)	25 (21%)	1 (20%)	0 (0%)	3 (33%)	
Preventive surgery (e.g., genetic mutation)	1 (1%)	0 (0%)	0 (0%)	0 (0%)	1 (11%)	
Simultaneous implantation of a breast implant						
Yes	93 (66%)	82 (70%)	5 (100%)	0 (0%)	6 (67%)	< 0.001
No	49 (34%)	36 (30%)	0 (0%)	10 (100%)	3 (33%)	
Duration of surgery (median [range] in minutes)^5^	117 (56–205)	119 (64–205)	108 (98–123)	79 (56–130)	116 (92–162)	0.009
Duration of hospital stay (median [range] in days)	7 (2–23)	7 (2–22)	4 (4–10)	9 (5–23)	6 (4–12)	0.002
Number of previous ipsilateral surgeries						
0	69 (49%)	64 (54%)	0 (0%)	5 (50%)	0 (0%)	< 0.001
1	33 (23%)	28 (24%)	1 (20%)	3 (30%)	1 (11%)	
2	27 (19%)	18 (15%)	3 (60%)	0 (0%)	6 (67%)	
≥ 3	13 (9%)	8 (7%)	1 (20%)	2 (20%)	2 (22%)	
Median (range)	1 (0–6)	0 (0–4)	2 (1–3)	0.5 (0–3)	2 (1–6)	
Previous ipsilateral radiation therapy						
Yes	17 (12%)	7 (6%)	3 (60%)	3 (30%)	4 (44%)	< 0.001
No	125 (88%)	111 (94%)	2 (40%)	7 (70%)	5 (56%)	
Ipsilateral radiation therapy after the procedure						
Yes	52 (37%)	46 (39%)	0 (0%)	6 (60%)	0 (0%)	0.013
No	90 (63%)	72 (61%)	5 (100%)	4 (40%)	9 (100%)	
Previous chemotherapy						
Yes	25 (18%)	13 (11%)	3 (60%)	6 (60%)	3 (33%)	< 0.001
No	117 (82%)	105 (89%)	2 (40%)	4 (40%)	6 (67%)	
Chemotherapy after the procedure						
Yes	69 (49%)	63 (53%)	0 (0%)	4 (40%)	2 (22%)	0.035
No	73 (51%)	55 (47%)	5 (100%)	6 (60%)	7 (78%)	

^1^Defined as breast reconstruction performed at the same time as mastectomy

^2^Defined as breast reconstruction following a previously performed mastectomy

^3^With or without placement of a new implant

^4^Either current diagnosis at time of procedure or previous leading diagnosis

^5^Total time between first skin incision and final wound closure, including all additional procedures

**Fig. 2:**
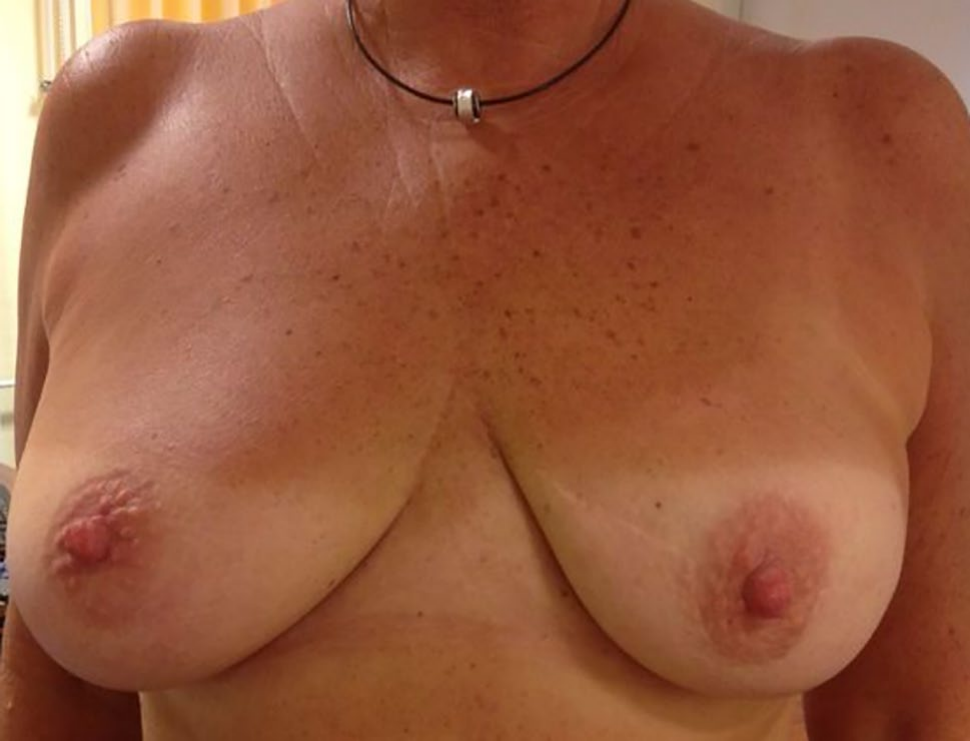
Postoperative result 12 years after a nipple-sparing mastectomy with complete removal of the skin of both lower quadrants and LDF-based direct-to-implant reconstruction of the left breast

**Fig. 3:**
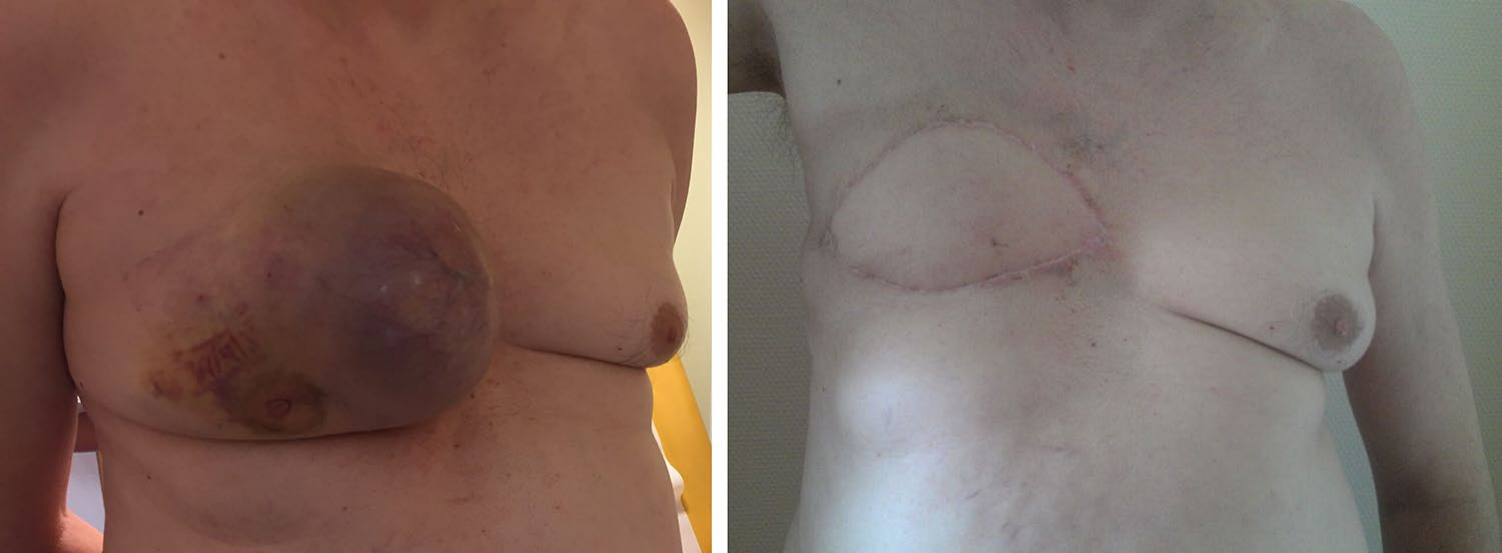
Pre- and postoperative view of a male patient with a large breast cancer receiving wide excision and LDF defect coverage

118 patients (83%) received immediate reconstruction, either with (n = 82) or without (n = 36) implant placement (Fig. 2, Table 1). The median volume of the inserted implant was 230 ml (range, 95–560 ml). In five patients, LDF surgery was performed for delayed reconstruction after mastectomy. Ten patients required LDF for defect coverage after removal of an advanced tumor (Fig. 3), and in nine cases LDF surgery was performed as revision procedure due to complications after previous surgery (e.g., implant-to-flap conversion in case of capsular fibrosis or wound healing disorders). Median duration of surgery, defined as time between first incision and final skin closure, was 117 min, including all additional procedures such as full axillary lymph node dissection (ALND, performed in 45 cases), sentinel lymph node biopsy (SLNB, 43 cases), implant removal and/or placement, removal of the port system, contralateral surgery, nipple reconstruction etc. The shortest duration of surgery was 56 min (83-year-old patient with breast cancer metastasizing to the bone receiving a palliative radical mastectomy for a large ulcerated breast cancer with defect coverage using an LDF flap). Comparing different clinical settings, the shortest median duration of surgery was observed in patients receiving LDF surgery for defect coverage (median 79 min). Patients remained in the hospital for a median of 7 days (range: 2–23 days). Most patients received at least one surgery of the ipsilateral breast prior to LDF procedure (range: 1–6). 12% underwent ipsilateral radiation therapy before and 37% after the surgery.

### Complications following LDF surgery

Observed complications are summarized in Table 2. The most common complication was a seroma occurring in 26% of procedures. 19% of patients received at least one needle aspiration for seroma (range: 1–6) and in one case revision surgery with an easy flow drain placement was necessary. Total flap loss due to complications was observed in three cases (2.1%). Revision surgery, defined as a second surgery for any reason within 30 days of LDF procedure, was performed in 13 out of 142 (9.1%) patients. Details on these patients are shown in Table 3. Previous application of chemotherapy did not affect complication rate, but previous ipsilateral radiation therapy was associated with significantly higher rates of infection (24% vs. 5%, p = 0.005). Lower rates of infection and wound dehiscence were observed in patients receiving primary reconstruction, compared to delayed reconstruction and defect coverage. Higher number of previous breast surgeries was associated with higher rates of nipple and/or skin necrosis (p = 0.010) and hematoma (p = 0.020).

**Table 2:** Complications following LDF surgery and their association with other factors

	Total	Seroma	Hematoma requiring surgical evacuation	Infection	Wound dehiscence	Partial skin and/or nipple necrosis	Flap loss due to necrosis
Total	142	37 (26%)	3 (2.1%)	10 (7%)	12 (8%)	10 (7%)	3 (2.1%)
Simultaneous implantation of a breast implant							
Yes	93	21 (23%)	2 (2.2%)	5 (5.4%)	7 (7.5%)	6 (6.5%)	2 (2.2%)
No	49	16 (33%)	1 (2.0%)	5 (10.2%)	5 (10.2%)	4 (8.2%)	1 (2%)
		p = 0.194	p = 0.966	p = 0.285	p = 0.586	p = 0.705	p = 0.966
Type of surgery							
Immediate reconstruction^1^	118	29 (25%)	3 (2.5%)	4 (3.4%)	8 (7%)	8 (7%)	2 (1.7%)
Delayed reconstruction^2^	5	2 (40%)	0 (0%)	1 (20%)	1 (20%)	0 (0%)	0 (0%)
Defect coverage	10	2 (20%)	0 (0%)	3 (30%	3 (30%)	1 (10%)	0 (0%)
Revision or implant-to-flap conversion^3^	9	4 (44%)	0 (0%)	2 (22%)	0 (0%)	1 (11%)	1 (11%)
		p = 0.492	p = 0.891	**p = 0.002**	**p = 0.044**	p = 0.861	p = 0.267
Previous ipsilateral radiation therapy							
Yes	17	6 (35%)	1 (6%)	4 (24%)	3 (18%)	1 (6%)	0 (0%)
No	125	31 (25%)	2 (1.6%)	6 (5%)	9 (7%)	9 (7%)	3 (2.4%)
		p = 0.355	p = 0.249	**p = 0.005**	p = 0.146	p = 0.842	p = 0.519
Previous chemotherapy							
Yes	25	7 (28%)	1 (4%)	3 (12%)	4 (16%)	0 (0%)	0 (0%)
No	117	30 (26%)	2 (2%)	7 (6%)	8 (7%)	10 (8%)	3 (2.6%)
		p = 0.807	p = 0.470	p = 0.286	p = 0.135	p = 0.129	p = 0.418
Number of previous ipsilateral surgeries							
0	69	17 (25%)	0 (0%)	4 (6%)	5 (7%)	2 (3%)	1 (1.4%)
1	33	7 (21%)	0 (0%)	1 (3%)	3 (9%)	1 (3%)	0 (0%)
≥ 2	40	13 (32%)	3 (8%)	5 (12%)	4 (10%)	7 (18%)	2 (5%)
		p = 0.513	**p = 0.020**	p = 0.247	p = 0.873	**p = 0.010**	p = 0.291

p-values < 0.05 are shown in bold

^1^Defined as breast reconstruction performed at the same time as mastectomy

^2^Defined as breast reconstruction following a previously performed mastectomy

^3^With or without placement of a new implant

**Table 3:** Details on patients receiving revision surgery

Patient number	Patient characteristics	Type of surgical revision	Follow up after revision
1	67-year-old patient receiving a mastectomy with immediate LDF + implant reconstruction for breast fibrosis after two breast-conserving surgeries and two radiation therapies (invasive breast cancer and invasive recurrence)	Hematoma evacuation	No further complications or sequelae
2	50-year-old patients receiving a mastectomy with LDF reconstruction for invasive breast cancer with positive margins following breast-conserving surgery and re-excision; ASA II	Hematoma evacuation	No further complications or sequelae
3	49-year-old patient with large DCIS with positive margins receiving a mastectomy with LDF + implant reconstruction after a breast-conserving surgery and re-excision	Hematoma evacuation and nipple removal for partial nipple necrosis	No further complications and normal wound healing
4	79-year-old patient receiving a radical mastectomy with defect coverage using a LDF for invasive angiosarcoma diagnosed 12 years after breast-conserving surgery and radiation therapy for breast cancer; ASA III with multiple comorbidities	Two minor surgical revisions for a back wound dehiscence (6 and 10 days after the LDF surgery)	Normal wound healing without sequelae
5	62-year-old patient receiving a delayed expander to LDF + implant reconstruction 2 years after mastectomy, chemotherapy, and radiation therapy; ASA II	Wide excision with flap and implant removal due to an unexpected invasive recurrence upon final histology and patient’s decision against reconstruction	Normal wound healing without sequelae
6	49-year-old patient with large DCIS receiving LDF + implant reconstruction for impaired wound healing and fistula following skin-sparing mastectomy and implant reconstruction; no previous irradiation or chemotherapy; heavy smoker, BMI 25, ASA II	Flap removal for flap necrosis (flap revision with implant removal on day 1 after LDF surgery and total flap removal 3 days later)	No further complications or sequelae
7	37-year-old patient with a multicentric invasive breast cancer receiving a nipple-sparing mastectomy with LDF + implant reconstruction and sentinel node biopsy; discharge from the ward on the 5th day after LDF surgery with normal wound healing; afterwards multiple appointments for ultrasound-guided aspiration of recurrent seroma	Placement of an “easy flow” drain for recurrent seroma 14 days after LDF surgery	No further complications or sequelae
8	68-year-old patient with a large invasive lobular carcinoma and LCIS receiving a mastectomy, axillary lymph node dissection level I-II and LDF + implant reconstruction	Minor surgical revision for a back wound dehiscence (26 days after the LDF surgery)	No further complications or sequelae
9	56-year-old patient with a large breast cancer with positive margins receiving a mastectomy with removal of the nipple-areola-complex and LDF + implant reconstruction following breast-conserving surgery and re-excision	Excision of skin necrosis with coverage using a small rotation flap 30 days after LDF surgery; no flap necrosis	No further complications or sequelae; contralateral reduction mammoplasty 7 months after LDF surgery for aesthetical reasons (asymmetry)
10	83-year-old patient with a large (18 cm) ulcerated breast cancer receiving a radical wide-excision mastectomy with defect coverage using a LDF; ASA III, multiple comorbidities	Minor revision with excision of skin necrosis and drain placement 14 days after LDF surgery	No further complications or sequelae
11	31-year-old patient with a large multicentric DCIS receiving a nipple-sparing mastectomy with LDF + implant reconstruction	Nipple removal 2 days after LDF surgery due to histopathological report (positive retromamillary margin); implant exchange and revision due to recurrent seroma with wound healing disorder 30 days after LDF surgery	No further complications or sequelae
12	61-year-old patient with a large invasive breast cancer receiving a mastectomy with LDF reconstruction for positive margins following breast-conserving surgery and re-excision; BMI 42, ASA III	Flap removal due to flap necrosis combined with expander placement 16 days after LDF surgery; flap perfusion in the first days after LDF surgery was normal	Normal wound healing; expander-to-implant surgery 6 months later
13	36-year-old patient with a multicentric invasive breast cancer receiving a mastectomy, sentinel node biopsy, axillary lymph node dissection level I and II and LDF + implant reconstruction; ASA II	Total flap loss with flap and implant removal 25 days after LDF surgery (initially normal wound healing with discharge from the ward on day 7)	No further complications during or after postoperative radiation therapy; patient does not wish a secondary reconstruction (follow up: 7 years)

*ASA* The American Society of Anesthesiologist Classification

### Follow up

The mean follow up was 38.4 months (range 0–200 months). None of the patients reported twitching or animation deformity of the flap. Out of 93 patients receiving an implant simultaneously with LDF surgery, 24 (26%) underwent implant removal. The median time of implant remaining in situ was 151.2 months (95% CI 90.8–211.6). The implants were mostly exchanged due to capsular fibrosis. In three cases the implant was permanently removed without replacement: in two cases the implant was removed due to invasive recurrence of the thoracic wall after 19 and 96 months, respectively, and in one case it was removed due to capsular contraction following radiation therapy without placement of a new implant (patient’s wish). The rate of implant removal and/or exchange in the long-term follow up was numerically higher in patients receiving pre- or postoperative radiation therapy but the difference was not statistically significant (32.4% vs. 21.8%, p = 0.256). The time point of radiation therapy was not significantly associated with implant removal and/or exchange (42.9% in case of radiation therapy before LDF surgery vs. 29% in case of postoperative radiation therapy).

## Discussion

To the best of our knowledge, this is the largest single-surgeon report on clinical characteristics as well as complications in patients undergoing latissimus dorsi flap-based surgery of the breast. In contrast to other publications, which mostly reported on delayed LDF reconstructions, the majority of patients in our study received LDF in an immediate reconstruction setting directly following a mastectomy. [7, 13].

The most common early complication was a seroma, occurring in 26% of patients. This is in line with previous reports and some authors suggested that asymptomatic seroma should not be considered a complication but rather an inevitable side effect of LDF surgery [11, 14]. The current practice of electrocautery dissection may further contribute to development of symptomatic or recurrent seromas, and it is not uncommon to leave drains for several days or even weeks at the donor site. Tomita et al. analyzed potential risk factors for seroma development in 174 patients with LDF surgery [15]. Seroma occured in 28% of patients receiving mastectomy and LDF, while the incidence was much lower (11%) in patients undergoing a breast conserving surgery with LDF-based volume replacement. Higher age and BMI significantly increased the risk of seroma formation. In our study, age (p = 0.045) but not overweight were correlated to seroma development. Different approaches to prevent seroma formation have been explored in previous studies. Lee et al. performed a meta-analysis of 14 studies, including three randomized controlled trials, on quilting sutures and fibrin sealants [16]. Both interventions contributed in varying degrees to reducing seroma-related morbidity following LDF transfer, and their combination might have a synergistic effect. However, more than half of the included studies had a retrospective and non-randomized design and the definition of seroma varied. Further, operative time required for either intervention, additional costs, and the potential for allergic reaction to sealants need to be considered. Therefore, neither intervention is accepted as standard-of-care and the decision to use them depends on surgeon’s preference. In the present study, the prevalence of seroma was comparable to that reported in previous studies, even though no intervention for seroma prevention was used. While drain placement is commonly used in LDF surgery, recommendations regarding the timepoint of drain removal vary. Some authors recommend removing the last drain when drainage volume decreased under a specific amount (e.g., 20, 30 or 40 ml) per 24 h, while others prefer to leave the drains for a defined time, irrespective of the output [17, 18]. In the present analysis, drains were removed once the drainage volume decreased to < 30 ml in 24 h.

The most serious complication in the setting of flap-based reconstructive procedures is a partial or total flap loss, usually occurring due to disrupted perfusion and subsequent necrosis. While total flap loss has been observed in 1–2% of patients across studies, partial flap necrosis may occur in up to 9% of cases [11] (Table 4). In our study, we observed a total flap loss rate of 2%. No patient suffered from partial flap ross. Thus, these results confirm LDF surgery as a safe and robust method with a low failure risk.

**Table 4:** Overview of complication rates following latissimus dorsi flap-based surgery reported in the literature (only publications on ≥ 100 procedures were considered)

Study	Number of flaps/patients	Complication rate (%)						
		≥ 1 complication	Total flap loss	Partial necrosis	Infection	Seroma	Reconstructive failure	Other observations
Our study	142/140	37%	2%	Flap: 0% Skin envelope and/or nipple: 7%	7% ^1)^	26%	2%	Implant exchange during follow up: 26%
Palve [13]	283/291	70%	0.3%	4%	3%^2^	50%	n.r	Shoulder morbidity: 7% Late implant-related complications: 15% (mostly capsular contracture)
Hardwicke [7]	243/277	n.r	0%	n.r	1.1%^2^	n.r	n.r	Capsular contracture Baker III with a necessity for a surgical intervention: 3.6% after a median follow up of 47 months Hematoma requiring surgery: 0.7% Further procedures not considered routine: 18%
Pinsolle [9]	n.a./201	52%	n.r	8%	1.5%	27%	2.5%	Capsular contracture: 7% Hematoma: 7%
Tomita [15]	174/174	n.r	n.r	n.r	n.r	21%	n.r	
Chang [10]	119/146	n.r	n.r	n.r	5%	n.r	13% ^3)^	Capsular contracture: 5.5% Implant rupture: 1.4% Implant loss: 7.5%
Paillocher [19]^5^	111/111	67%	n.r	5%	2%	54%	n.r	Hematoma: 4%
Daltrey [14]	106/106	n.r	n.r	n.r	n.r	89%^4^	n.r	
Houvenaeghel [20]	105/105^2^	55%	n.r	n.r	2%	33%	n.r	Hematoma: 3% Reoperation: 3%
Giacalone [21]	104/104	58%	0%	Marginal flap necrosis: 9% Marginal back skin necrosis: 9% Breast skin envelope necrosis: 5%	5%	17%	5%	Hematoma: 13% Capsular contracture: 13% ≥ 1 late complication: 24% Implant revision: 26% Back pain 7%
Sternberg [8]	100/100	n.r	0%	Flap: 1% Mastectomy skin flap necrosis: 8%	n.r	34%	n.r	Capsular contracture: 16% Surgical correction for capsular contracture: 6% Revision for aesthetic reasons: 10% Donor site wound problems: 9%

^1^Including minor infections and those treated by conservative means

^2^Only infections leading to implant removal were considered

^3^Defined not as permanent reconstructive failure and partly treated with implant exchange

^4^In this randomized study a specific suture technique was used for seroma prevention (seroma rate 83% vs. 96% in control arm)

^5^All patients received neoadjuvant chemotherapy and radiation therapy directly before LDF surgery

Arm and shoulder morbidity and—in case of a simultaneous implant placement—capsular contracture, are considered typical late complications of LDF based surgery. In the setting of immediate or delayed reconstruction, LDF usually requires a combination with an implant to achieve desired breast size, due to the limited volume of the flap itself. In the present study, 26% of patients who received an implant required implant removal during follow-up. In most cases, this was followed by an immediate placement of a new implant (implant exchange). The reported implant removal or exchange rate in studies with longer follow-up may be as high as 50% [22–24]. Rates on the prevalence of capsular fibrosis following combined LDF and implant reconstruction vary widely among studies. Hardwicke et al. examined 277 consecutive LDF procedures combined with a placement of a textured cohesive gel implant, focusing on late complications [7]. After a mean follow-up of 47 months, higher-grade capsular contracture, defined as Baker III or IV, occurred in 3.6% of patients, resulting in a capsulotomy in all patients. Interestingly, administration of a chemotherapy was associated with a lower rate of capsular contracture, while no association with radiation therapy was seen. Similarly, in our analysis the administration of radiation therapy was not associated with implant removal rate in the long-term follow up. The mean time to first implant exchange was 22.4 months and thus shorter than in the present study.

Giacalone et al. reported on the long-term clinical follow-up of 104 patients in two cohorts: 26 patients received an immediate reconstruction using a LDF and prosthesis (either a tissue expander or a permanent implant) after neoadjuvant chemotherapy and 78 a delayed reconstruction after chemo- and radiation therapy [21]. Early implant loss occurred more frequently in the delayed reconstruction setting (12% vs. 0%), whereas late complications were more common after immediate reconstruction (30% vs. 21%). Capsular contracture rates were similar in both cohorts (15.3% after immediate and 11.5% after delayed reconstruction, respectively). The prevalence of capsular contracture increases with time [24].

In the present work no arm and shoulder-related problems were documented after a median follow-up of 38 months. However, data on shoulder impairment were not prospectively collected and no measurements of shoulder strength performed. Therefore, underreporting cannot be excluded. Altogether, data on arm and shoulder morbidity after LDF surgery vary across studies [11, 24, 25]. According to a meta-analysis of 26 studies, there is a tendency that the LDF transfer may affect shoulder function, but this limitation seems to be minimal, and few patients experience a major impact on shoulder function [26].

Regarding the surgical technique, two aspects need to be considered. First, it has been shown that transection of the tendinous insertion of the latissimus dorsi muscle on the humerus may improve aesthetic results [27]. Specifically, it helps to avoid the development of the displeasing bulge that patients often refer to as “carrying a book” under the armpit and may increase the mobility of the flap [27]. In the present study, the humeral insertion of the muscle was transected in all cases. Second, transecting the thoracodorsal nerve has been controversially discussed in the literature [12, 28–31]. In the present study, a simple transection of the thoracodorsal nerve was performed in all patients, which reflects the standard approach followed by most surgeons in Germany [27, 28, 32]. While leaving the nerve intact may lead to unintentional muscle twitching and breast animation, sometimes referred to as “jumping breast” phenomenon, some authors suggested that cutting the nerve may result in a decrease of flap volume and proposed an individual approach depending on the setting and the complexity of nerve identification [29, 30]. Interestingly, several studies reported that reinnervation may occur after simple nerve transection and that reinnervation rates may be reduced when larger nerve segments are resected [30].

The present study shows a slow decrease in the use of LDF-based breast reconstruction. A similar trend is currently being observed world-wide. Leff et al. examined immediate breast recontructions performed in the United Kingdom [33]. After an initial increase in the use of the technique observed between 1996 and 2008, the number of procedures decreased rapidly. At the same time, the use of other techniques of autologous tissue transfer increased from 0.44% in 1996 to 2.76% in 2012. A similar trend was noted in the United States as well [34–36]. Various reasons have been proposed to explain the decreased use of LDF procedures. First, the relatively low volume of the harvested flap makes a simultaneous use of a breast prosthesis necessary in most cases, which makes the technique unsuitable for patients opting for an implant-free reconstruction. Second, a worldwide trend towards bilateral mastectomy is observed [37, 38]. While LDF-based reconstruction of both breasts is possible, arm and shoulder morbidity after a bilateral procedure remains a concern. Finally, the improved access to abdominal flap transfer with microvascular anastomosis at high-volume centers makes pedicled flaps less attractive.

## Conclusions

The present study confirms latissimus dorsi flap to be a safe and robust method for reconstruction of the breast and skin replacement of chest wall. As in previous studies, the leading early complication was a seroma, while serious complications such as total flap loss were a rare occurrence. Future studies should focus on the long-term evaluation of arm and shoulder morbidity as well as patient satisfaction with the aesthetic outcome.

### Supplementary Information

Below is the link to the electronic supplementary material.

Supplementary file1 (DOCX 14 KB)
