# Supplementary material for: Latissimus dorsi flap for breast reconstruction: a large single-institution evaluation of surgical outcome and complications
Source: Arch Gynecol Obstet. 2023 Aug 16;309(1):269–80. doi: 10.1007/s00404-023-07186-3 (PMC10770241; doi:10.1007/s00404-023-07186-3)
Supplement: Supplementary file 1 — Supplementary file1 (DOCX 14 KB) [file 404_2023_7186_MOESM1_ESM.docx]

**Supplementary Table 1.** Number of LDF-based surgeries per year in the period analyzed (2005 – 2022).

| **Year** | **Number of LDF-based procedures** | **Percentage of LDF-based procedures in relation to all breast cancer patients treated** |
| --- | --- | --- |
| 2005 | 15 | 7,0% |
| 2006 | 12 | 5,6% |
| 2007 | 14 | 6,6% |
| 2008 | 16 | 7,5% |
| 2009 | 5 | 2,3% |
| 2010 | 9 | 4,2% |
| 2011 | 8 | 4,3% |
| 2012 | 3 | 1,3% |
| 2013 | 10 | 4,2% |
| 2014 | 8 | 3,6% |
| 2015 | 8 | 3,6% |
| 2016 | 6 | 2,9% |
| 2017 | 7 | 3,6% |
| 2018 | 5 | 2,0% |
| 2019 | 4 | 1,3% |
| 2020 | 2 | 0,6% |
| 2021 | 6 | 1,7% |
| 2022 | 4 | 1,1% |
| Total 2005-2022 | 142 | 3,2% |
